# Increased Flight Altitudes among Migrating Golden Eagles Suggest Turbine Avoidance at a Rocky Mountain Wind Installation

**DOI:** 10.1371/journal.pone.0093030

**Published:** 2014-03-26

**Authors:** Naira N. Johnston, James E. Bradley, Ken A. Otter

**Affiliations:** Ecosystem Science and Management, University of Northern British Columbia, Prince George, British Columbia, Canada; University of Regina, Canada

## Abstract

Potential wind-energy development in the eastern Rocky Mountain foothills of British Columbia, Canada, raises concerns due to its overlap with a golden eagle (*Aquila chrysaetos*) migration corridor. The Dokie 1 Wind Energy Project is the first development in this area and stands as a model for other projects in the area because of regional consistency in topographic orientation and weather patterns. We visually tracked golden eagles over three fall migration seasons (2009–2011), one pre- and two post-construction, to document eagle flight behaviour in relation to a ridge-top wind energy development. We estimated three-dimensional positions of eagles in space as they migrated through our study site. Flight tracks were then incorporated into GIS to ascertain flight altitudes for eagles that flew over the ridge-top area (or turbine string). Individual flight paths were designated to a category of collision-risk based on flight altitude (e.g. flights within rotor-swept height; ≤150 m above ground) and wind speed (winds sufficient for the spinning of turbines; >6.8 km/h at ground level). Eagles were less likely to fly over the ridge-top area within rotor-swept height (risk zone) as wind speed increased, but were more likely to make such crosses under headwinds and tailwinds compared to western crosswinds. Most importantly, we observed a smaller proportion of flights within the risk zone at wind speeds sufficient for the spinning of turbines (higher-risk flights) during post-construction compared to pre-construction, suggesting that eagles showed detection and avoidance of turbines during migration.

## Introduction

The construction of wind-energy installations in areas regularly used by raptors raises concerns over potential collision fatalities [1], [2]. Preliminary findings suggest that raptors are at lower risk of collision fatality with turbines during migration [3], [4] (although see [5]), compared to breeding and overwintering populations that spend more time in the area of a development [6]–[8]. It is difficult to conclude whether lower collision rates are due to detection and avoidance, however, because few before-after construction studies have documented species-specific flight behaviour in response to the placement of wind-energy developments along raptor migration corridors [9], [10]. Further compounding the issue is a lack of research on how raptors respond to the new, taller models of wind turbines currently being employed [10], [11].

Soaring raptors rely on wind for lift to reduce energetic costs during migration. This can be in the form of vertically-rising air that results from either wind being deflected by underlying topography (orographic lift) or thermals (rising warm air) [12]–[14]. Heavy-bodied species, such as golden eagles (*Aquila chrysaetos*), rely upon soaring flight to conserve energy during migration, which may place birds at risk of colliding with man-made structures during conditions that are not favorable for gaining altitude [4], [15]. For example, in Spain, resident Griffon Vulture (*Gyps fulvus*) collisions with turbines increased under weak-wind conditions along gentle slopes. Under such non-lift generating wind conditions, birds were forced to gain altitude by using slow circle-soaring flight on thermals, often in airspace that overlapped with turbines [4]. Further, some topography features of ridgelines generate vertically-deflected air (orographic lift) that provide sources of lift to soaring birds – depending upon the altitude of birds traversing such features, wind development on these same ridges could be associated with higher collision potential for raptors [3], [4], [16]–[19].

During periods with high wind speeds, particularly when migration direction is oriented into a headwind, soaring raptors often fly closer to the underlying topography [14], [20], [21]. Strong winds reduce the availability and creation of thermals, forcing birds to rely upon orographic lift. In rugged environments, such as mountainous terrain, orographic lift is often strongest in the airspace within several hundred meters above the underlying topography, which could increase the chance of migrants flying at heights corresponding to turbine locations.

Displacement, or complete avoidance, of a wind-development area by raptors has been noted as a response to wind development [9], [22], [23]. Displacement can also occur, however, on a micro-scale, where avoidance is associated with the turbines themselves through small-scale changes in flight patterns [23]–[28]. While repeated flight paths of resident birds within such areas may lead to habituation to turbines and potential collisions with blades [24], [29], micro-scale avoidance by birds traversing such a site briefly once or twice a year on migration may result in low collision risks. As an example of this, Johnson et al. [30] found evidence to suggest that siting turbines away from the main slopes used by migrating golden eagles in Wyoming resulted in fewer fatalities than expected from pre-construction flight behaviour. Whether certain weather conditions and/or ridge-top features place migrating golden eagles at greater risk of colliding with turbines, however, is not known.

In North America, golden eagles migrate in large concentrations along relatively narrow corridors of the major mountain ranges, including the eastern Rocky Mountains [31]–[33]. The thrust-fault formation of the Rocky Mountains, particularly in Canada, results in ridges and foothills aligned in a consistent southeast to northwest direction [34], which in many areas along its length, is perpendicular to the prevailing winds. This creates strong and consistent updrafts along the north-south length of the range, serving as an aerial highway for migrating golden eagles in the fall [31]–[33]. One section of this flyway in British Columbia (BC), Canada (the Hart Range), has been identified by the Provincial government as having strong development potential for wind energy [35]. The first commercial wind installation under construction in the Hart Range - the Dokie 1 Wind Energy Project - has characteristic topographic orientation and exposure to weather patterns, including wind strength and direction, for the range [32], [33]. Like most of the sites in the region under consideration for wind development, it also falls within the migratory corridor of golden eagles.

Our purpose was to document pre- and post-construction golden eagle flight altitudes in proximity to the Dokie I Wind Energy Project. Although the ability of a golden eagle to detect a turbine during migration is expected to be high [36], we were interested in determining how eagles respond to the presence of turbines and whether certain weather conditions and/or ridge-top topography features (either sloped or flat sections) place eagles at lower altitudes and thus at greater risk of collision. We collected data over one pre-construction, and two post-construction fall seasons to determine whether golden eagles adjust their behaviour to the presence of wind turbines, and, if such adjustments were weather and/or ridge-top topography dependent. We: 1) compared between years the proportions of eagles that flew within a 100 m ridge-top area around the location of the turbine string at heights corresponding to the rotor-swept area (risk zone; <150 m above ground), as well as what proportion of these flights were at wind speeds when the turbines would have been spinning (higher-risk); and, 2), identified the extent to which the probability of golden eagle flight altitude responded to weather (wind direction, wind speed, cloud cover and temperature) and/or ridge-top topography. Although our study focuses on only a single wind development, future developments in the region are currently being constructed along topographically-similar ridges that experience similar weather patterns. Therefore, our results are widely applicable and could be used to predict collision risk associated with future development throughout the Hart Range and eastern Rocky Mountains.

## Methods

We would like to acknowledge and thank the West Moberly First Nations, Halfway River First Nation, Saulteau First Nations and McLeod Lake Indian Band for supporting our activities in their territory.

**Figure 1:**
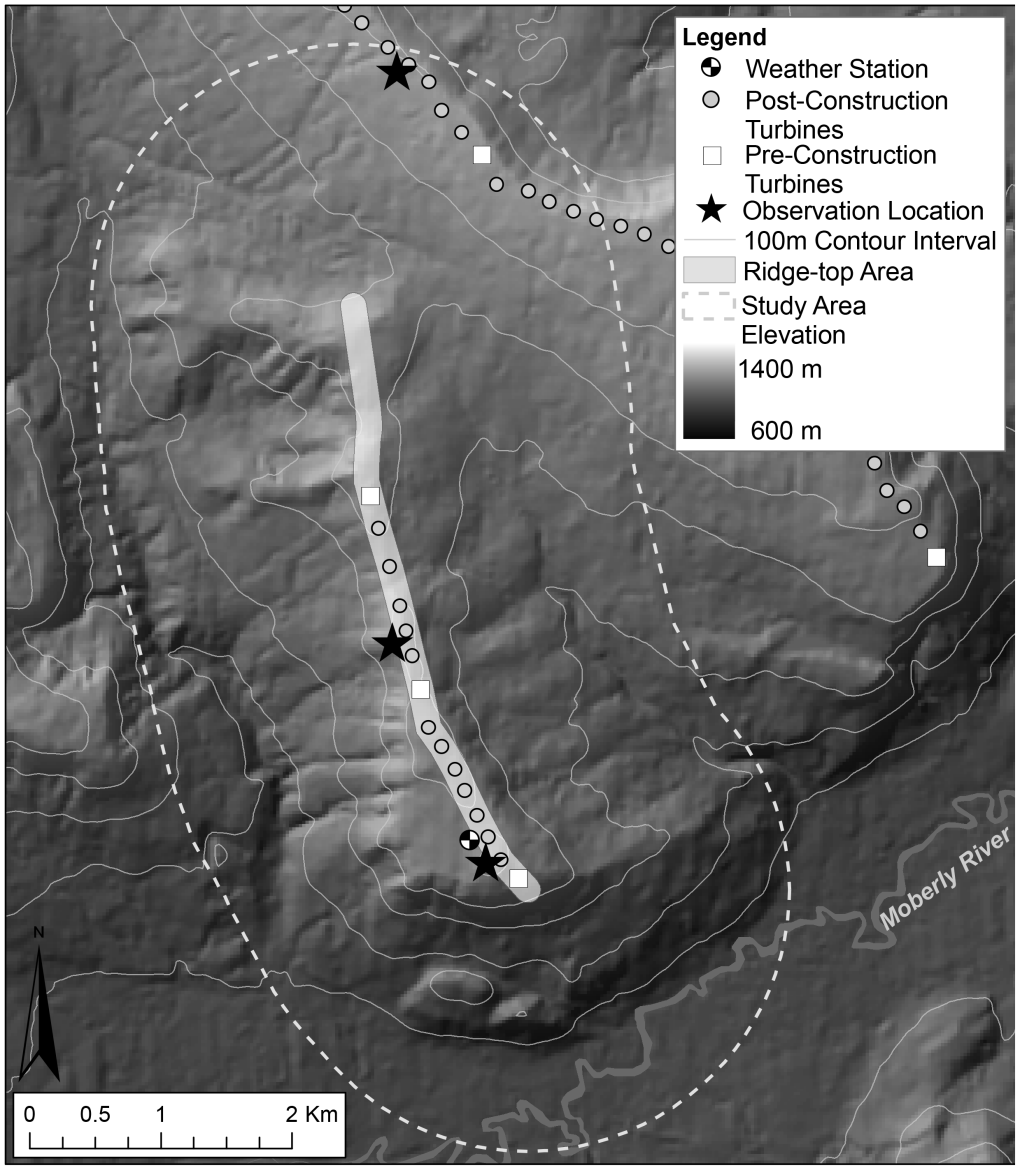
Study site. Site map of the Dokie 1 Wind Energy Project in the Peace River Regional District of northeast British Columbia, Canada (55°46′ 28″ N, 122°16′ 49″ W).

We documented golden eagle flight tracks as they migrated through the Dokie 1 site (55°46′28″N, 122°16′49″W) between 30 September –24 October in 2009, 2010, and 2011. Observations were conducted between 0900 and 1530 Pacific Daylight Time each day and were divided into two three-hour time blocks (am or pm). We surveyed every day, weather and visibility permitting, for a total of 28.5 days (111 hours) in 2009, and 28 days (108 hours) in both 2010 and 2011. We focused our observations on the shorter (4.5 km) and lower elevation (1200 m) ridgeline associated with this development due to previous observations that eagle movements were concentrated along this ridge [37], in addition to the greater availability of observation points that allowed a panoramic view of both the ridge-top area and the surrounding valleys (see [38]). Observers worked in pairs, one estimating eagle positions while the other recorded and kept track of individual birds. The same observers (NNJ & JEB) were used in this study in all years. One observer estimated eagle locations in all three years (JEB), thus reducing error introduced by observers, and making the error that did exist consistent within and between years [24]. Three observation locations were used to ensure full coverage of the study ridge. Observations were made sequentially from two locations per day and were rotated between days to account for time (am or pm). Two observation locations were on the study ridge, and one was on an adjacent ridge (Fig. 1).

We considered 2009 as pre-construction despite three widely-spaced idle turbines (Vestas 3-MW; 127 m tall to tip of blade) standing 1.5 km apart. No construction or site activity occurred in this year. We considered 2010 and 2011 as post-construction due to the presence of a full string of turbines along the ridge-top. By the fall of 2010, 15 turbines were erected on the study ridge but were not powered or spinning. Construction activity was heavy and testing of turbines occurred near the end of the survey period. Construction was finished, and all turbines were functional before fall 2011. Although 2010 could be considered as a construction year rather than a post-construction year, we included 2010 data to compare the reaction of migrating golden eagles to the presence of densely-spaced turbines along a ridge-top. Hence whether or not turbines were spinning is not considered in this study.

The north end of the study ridge, 1.5 km in length, was not developed, but was included in our study as a control area (3 km in length). Since research suggests that the placement of turbines at ridge ends may increase collision risk [17], including the entire ridgeline allowed us to assess whether eagle flights were at lower elevations over particular topographic aspects of the ridge.

We collected three-dimensional locations of eagles as they migrated through the study site, or within 2 km of the observer from our observation locations [38]. Eagle locations were estimated in relation to the position of the observer; we used a compass for the relative bearing to the bird, and estimated distance to the bird using the help of maps with distance rings and known landmark features. The average distance that eagles were detected crossing the ridge-top area was less within 1 km (760±63 m) from the observer [38]. Eagle altitude relative to the observer was estimated using an inclinometer measured to 0.5 degrees. We collected data points upon first sighting of the bird within the 2 km focal area, and collected between six-to-twelve sequential point locations per bird, depending on the proximity of its approach to the ridge-top area, as it migrated through the site [38]. The average time that an eagle was tracked through the site (within 2 km from the observer) was approximately 2.5 minutes (median: 2 min 15 sec; minimum: 13 sec; maximum: 10 min 53 sec). From these sequential measurements, eagle positions were converted to UTM coordinates with respect to the observer location using trigonometry on bearing and distance in Excel (see [38]). Elevations associated with each point location were calculated using inclinometer angles and the bird’s position relative to the observer. Only birds that travelled in a south-bound direction were included. Hence, the study area refers to eagles observed within 2 km of an observation point, spread out over three locations (Fig. 1).

We converted sequential UTM locations for each bird into individual flight tracks using ArcGIS (Environmental Systems Resource Institute, 2009. ArcMap 9.3, Redlands, California), and elevation of each sequential point along the track was input to create 3-Dimensional flight lines (ET Spatial Techniques, Pretoria, South Africa, www.ian-ko.com) [38]. From these tracks, we identified golden eagles that flew over the ridge-top area – this was defined as having crossed into a 100 m area surrounding the turbine string along the top of the ridge (see [38]; Fig. 1). Tracks that entered this area were intersected at the point of entry and a flight altitude was extracted for that location (ET Spatial Techniques). Eagle flight altitudes were then subtracted from the elevation of the terrain directly below the point using information from a 50×50 m digital elevation map to obtain a flight height (m above ground [ag]; herein called flight altitude). For eagles that crossed over the ridge-top area multiple times, only the first point of entry was used in order to identify conditions, and/or locations, under which eagles first approached the ridge-top area. We identified eagles that crossed the ridge-top area as having entered the “risk zone” when their height was within 150 m of the ground. Although the height of a turbine is 127 m to the tip of the blade, we used 150 m to allow for errors in distance, and thus height estimations [38]. All movements within the ridge-top area were classified equally. For example, we did not differentiate between movements parallel to the turbine string versus crossing the turbine string, or movements between adjacent turbines that fell within or just beyond the rotor-swept area of either tower.

We used an Onset HOBO weather station (Onset, Bourne, MA, USA) to collect ground-based data every five minutes at the south end of the study ridge. In 2011, we obtained wind data collected at nacelle height (80 m) from the closest turbine to our weather station to estimate the increase in wind speed with increasing altitude to better assess turbine cut-in speed at nacelle height from ground-based wind speed data. A ground-based wind speed at 2 m above ground of 6.8 km/h was correlated using linear regression to a 14.4 km/h wind at nacelle height, the cut-in speed at which turbines begin rotation (80 m ag; Ground-based wind speed = 0.3836+0.4436 [nacelle height wind speed]; *R^2^* = 0.88). We considered higher-risk movements to occur when birds were both in the risk zone (≤150 m ag) and wind speeds exceeded turbine cut-in speeds (≥6.8 km/h ground speed).

We created wind-direction classes based on orientation relative to the western side of the study ridge, as this was the route the majority of eagles used to travel through the site [38]. A headwind consisted of a direction originating from 136–225°, a western crosswind from 226–315°, a tailwind from 316–45°, and an eastern crosswind from 46–135°. We considered the categorization of wind direction measurements a more practical approach to dealing with multicollinearity between wind speed and direction [39]. We confirmed our use of wind direction categories by running a separate model omitting 21 data points that were within 2 degrees of our directional cut-off between headwinds and western crosswinds. Model results did not change, which suggested that our model was not overly influenced by the inclusion of the 21 data points.

We created three eagle-age categories to account for the difficulty in separating some of the field marks between classes; *young* birds (juvenile and sub-adult individuals), *adult* birds and *unknown age* [40].

### Statistical Methodology

We used logistic regression to analyze the probability of flights over the ridge-top area that were within the risk zone (≤150 m ag), or not (>150 m ag). We considered the following predictor variables: topography type (slope or flat area); weather variables: wind speed (km/h), wind direction category (head, cross or tailwind), temperature (°C), relative humidity (%), and cloud cover (%); turbine or control area; construction phase (pre or post); date; hour; and age (adult, young or unknown age). We tested the degree of correlation between independent variables prior to model selection, and retained the most significant variable in further models. However, we substituted the alternative variable to verify that we had chosen the best representation of the effect. We used backward step-wise model selection criteria using Akaike’s Information Criterion (AIC) values to compare the final models, and considered all models within a ΔAIC of 2 [41]. The final model was chosen based on the principal of parsimony. We tested our final model against a null model using a likelihood ratio test [42], and used 10-fold cross validation to obtain an estimate of predictive accuracy (Maindonald and Braun 2009). We included interactions between independent variables that could potentially have influenced eagle heights (i.e. wind speed and temperature, wind speed and wind direction category).

We used chi-squared goodness-of-fit tests to compare annual numbers of eagles observed at the site to the number of corresponding ridge-top crosses, risk-zone crosses (≤150 m ag) and higher-risk crosses (≤150 m ag and winds ≥6.8 km/h ground speed) observed [43].

We used R (version 2.8.1, R Foundation for Statistical Computing, Vienna, Austria) for all analyses and Oriana version 2.0 (Kovach Computing Services, Anglesey, Wales, UK), to plot circular wind-direction data.

## Results

**Figure 2:**
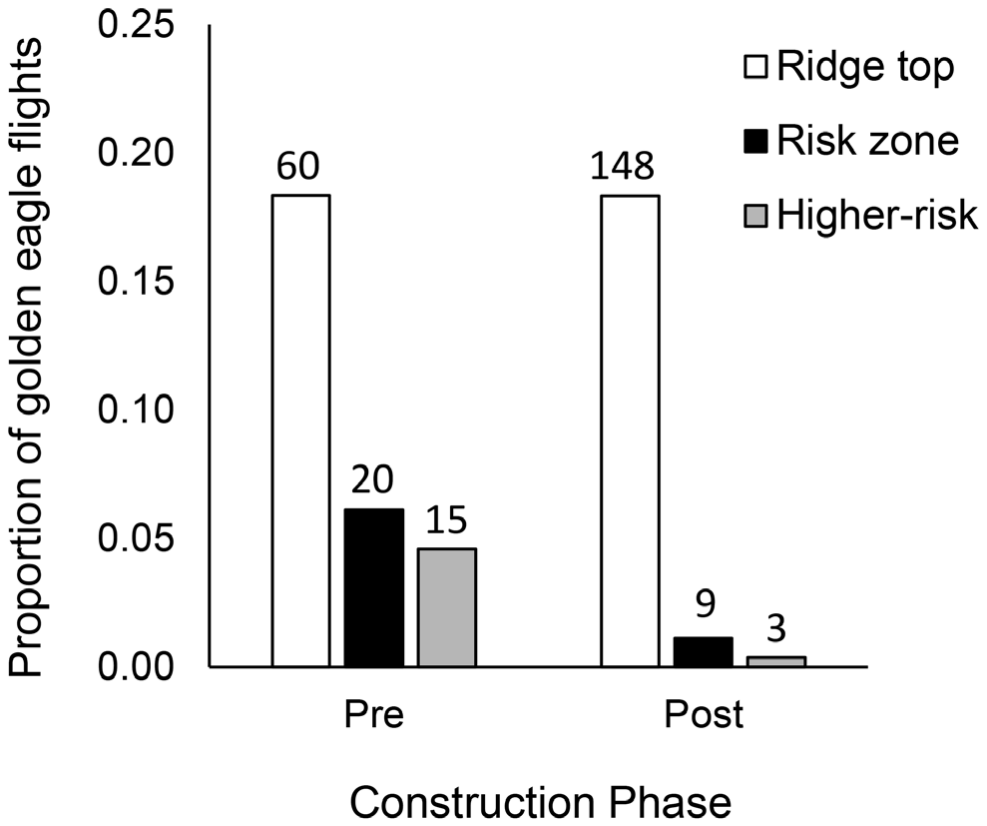
Proportion of golden eagle risk zone and higher-risk crosses. Proportion of golden eagles observed in the study area that flew over the ridge-top area (100 m buffer around proposed turbine string), at heights considered to be within the turbine risk zone (≤150 m above ground), or made a higher-risk flight into the risk zone (risk zone crosses that occurred at winds above turbine cut-in speed [6.8 km/h]) during pre-construction versus post-construction years. Values above bars represent sample size.

A total of 1134 golden eagles were tracked within the study site during three fall migration seasons (2009, *n* = 327; 2010, *n = *380; 2011, *n* = 427), with a greater number counted in post-construction (2010 and 2011) compared to pre-construction (2009; Table 1). Although our sample size for the total number of eagles that passed through the site was higher in each of the post-construction years, the proportion of crosses over the ridge-top area – regardless of flight altitude – did not differ between years (18%; Table 1; Fig. 2). We did, however, observe a significantly smaller proportion of crosses into the risk zone (within rotor-swept height; ≤150 m ag) in post-construction years (1%) compared to pre-construction (6%; Table 1; Fig. 2). Furthermore, not all of the crosses into the risk zone involved instances of higher-risk flights - where wind speeds were sufficient to spin the turbine blades (turbine cut-in speed; ≥6.8 km/h ground speed). Here, we observed a smaller proportion of higher-risk crosses into the risk zone in post-construction (<1%) compared to pre-construction (5%; Table 1; Fig.2).

**Table 1:** Percent of all golden eagles observed in the study site (within 2 km from turbine string) that were: over the ridge-top area (within 100 m from turbine string); within the risk zone (≤150 m above ground); and, within the risk zone at winds above turbine cut-in speed (higher-risk flight; 6.8 km/h) at the Dokie 1 Wind Energy Project site between 30 September–24 October in pre- (2009) and post-construction (2010–2011) years.

	Pre-		Post-			
	%	*n*	%	*n*	X^2^	*P*
Site (2 km)	29	327	71	807	–	–
Ridge-top cross	18	60	18	148	0.01	0.92
Ridge-top cross within risk zone	6	20	1	9	26.45	<0.01
Higher-risk cross	5	15	0.004	3	25.67	<0.01

We found the likelihood for a golden eagle to cross over the ridge-top area within the risk zone (≤150 m ag) increased by: 6 times during pre- versus post-construction years; 2.5 times under headwinds; and 8 times under tailwinds vs western crosswinds (LR-test χ_1_^2^ = 60.60, *P*≤0.001; cross-validation estimate of predictable accuracy = 87%; Table 2). Conversely, we found risk zone crosses decreased by 13 percent per kilometer increase in wind speed (Table 2). The probability to cross over the ridge-top area at turbine height did not differ between golden eagle age categories (adult versus sub-adults and juveniles), ridge-top topography features (slopes versus flat areas) or between the 1.5 km long control area compared to the 3 km area with turbines.

**Table 2:** Summary of logistic regression examining the association of temporal and environmental variables on the likelihood of flying over the ridge-top area (100 m buffer around proposed turbine string) at rotor swept height (risk zone; ≤150 m above ground) at the Dokie 1 Wind Energy Project between 30 September–24 October, 2009–2011.

Term	Estimate	SE	*z*	*P*
Intercept	−1.809	0.748	−2.418	0.016
Pre vs Post–Construction	1.971	0.521	3.785	≤0.001
Wind Speed (km/h)	−0.139	0.044	−3.144	0.002
Wind Direction – East Crosswind	17.24	1385	0.012	0.990
Wind Direction – Headwind	1.219	0.570	2.138	0.032
Wind Direction – Tailwind	2.198	0.897	2.450	0.014

The response measure under consideration was whether eagles flew within the risk zone (1) or not (0).

**Figure 3:**
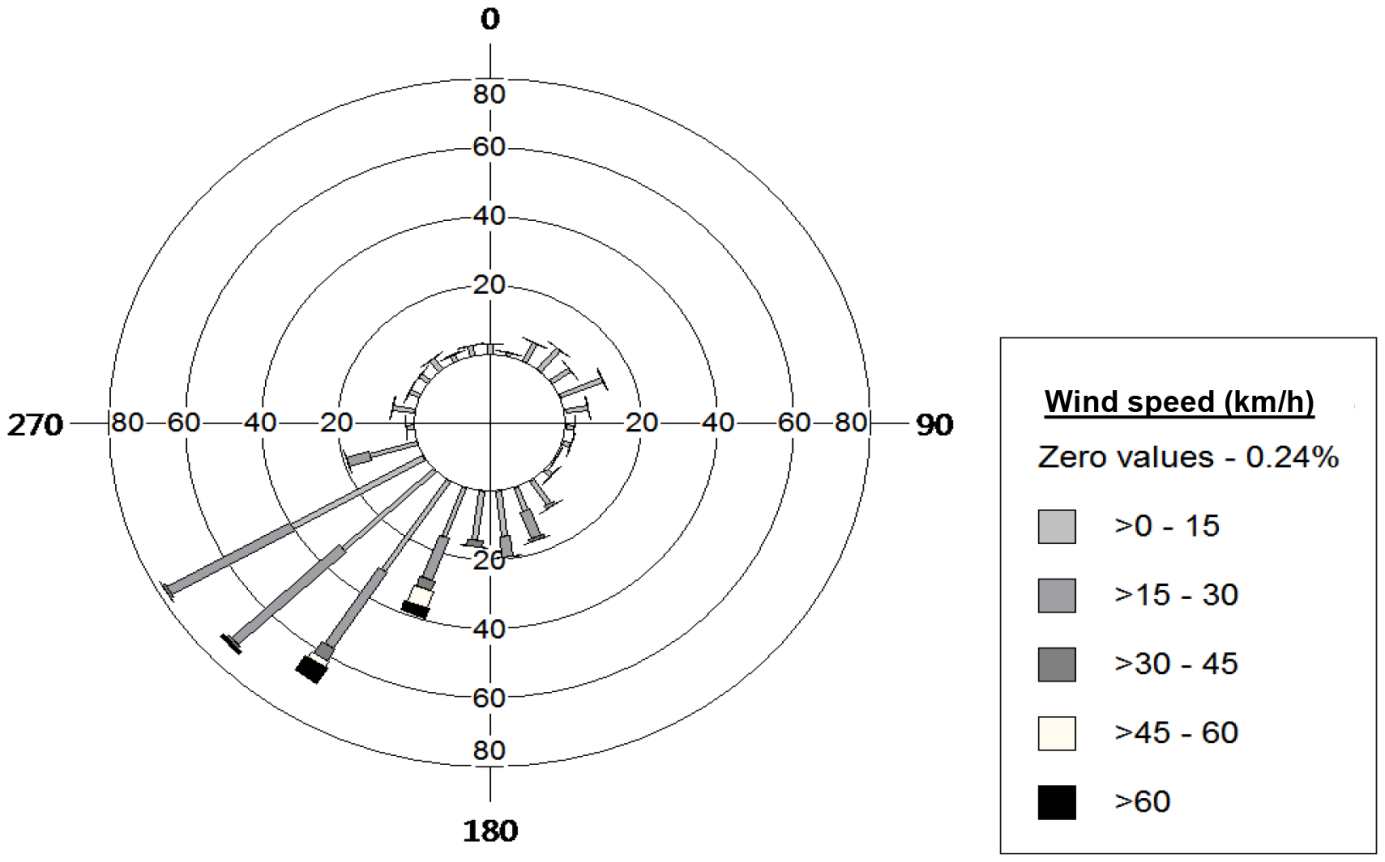
Wind direction (°) by wind speed (km/h). Ground-based wind direction and wind speed for observation hours (417 hours) over three fall migration seasons between 30 September –24 October, 2009–2011 at the Dokie 1 Wind Energy Project, BC, Canada.

**Figure 4:**
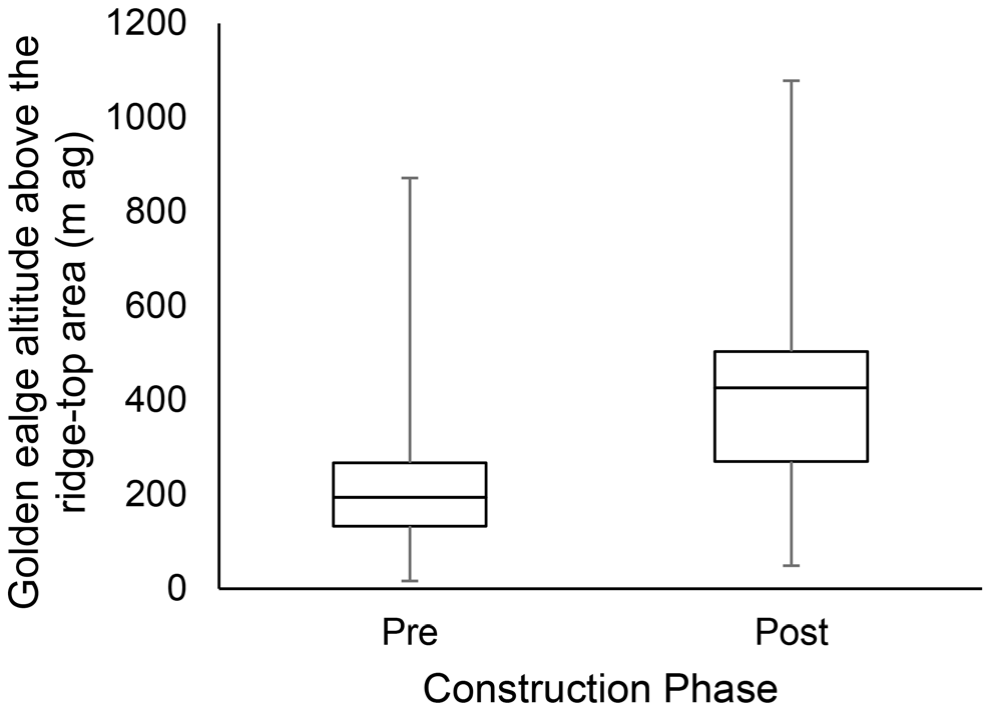
Golden eagle flight altitude (m above ground) above ridge top. Golden eagle flight altitudes above the ridge-top area during fall migration over one pre-construction (*n* = 60) and two post-construction (*n* = 148) seasons. Box represents median, first and third quartiles, and whiskers the maximum and minimum altitudes. Dashed line represents risk zone (≤150 m above ground).

**Figure 5:**
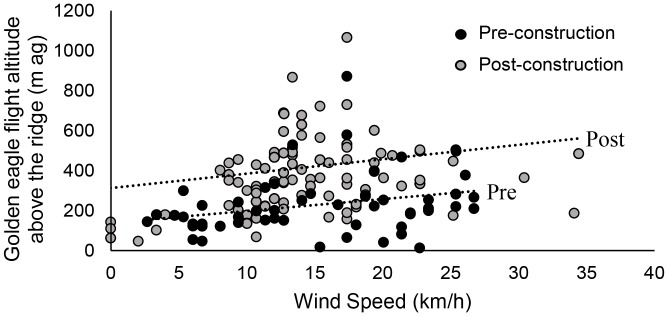
Golden eagle altitude (m above ground) over ridge top by wind speed (km/h). Golden eagle flight altitude above the ridge-top area (m above ground) versus ground-based wind speed (km/h) during pre- (*n* = 60) and post-construction (*n* = 148) years. Some data points overlap. Grey box represents higher-risk flight zone (risk zone [≤150 m above ground] and above turbine cut-in speed [6.8 km/h]).

Prevailing southwest winds, comprised of headwinds and western crosswinds, occurred in equal proportions in pre- and post-construction (approx. 45% each; Fig. 3). On average, winds were stronger in post-construction compared to pre-construction (median and 90^th^ Percentile for pre- and post-construction respectively: 7.9, 21.0 km/h; 15.1, 30.6 km/h). Golden eagle flight altitude (m ag) above the ridge-top was higher in post-construction compared to pre-construction (median and 90^th^ Percentile for pre- and post-construction respectively: 189, 396 m; 404, 697 m; Fig. 4), and while accounting for differences in wind speed (Fig. 5).

**Figure 6:**
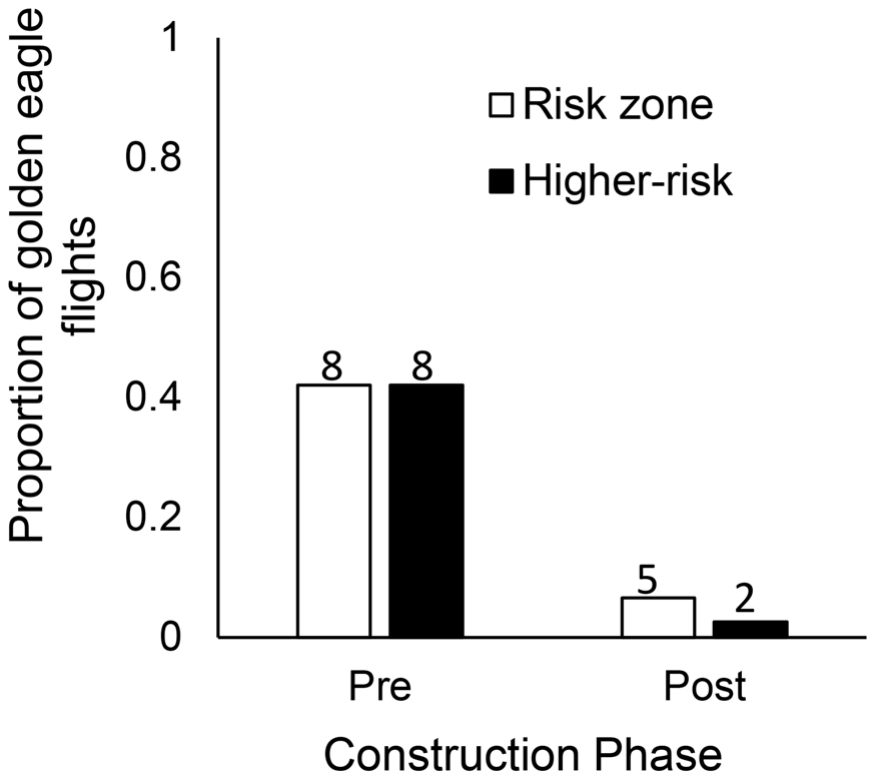
Proportion of risk zone and higher-risk crosses under headwinds. Proportion of golden eagle ridge-top crosses within risk zone (≤150 m above ground) and of higher-risk (within risk zone at winds above turbine cut-in speed 6.8 km/h) under headwind conditions during pre- and post-construction. Values above bars represent sample size.

During pre-construction, over fifty percent (*n* = 31) of all crosses over the ridge-top area occurred under western crosswind conditions, however, only 13% (*n* = 4) occurred within the risk zone (≤150 m ag). By comparison, a third (*n* = 19) of ridge-top crosses occurred under headwinds, but represented over 42% (*n* = 8) of all crosses within the risk zone. Of the eagles that crossed into the risk zone under headwinds, all (*n* = 8) were under higher-risk conditions when the turbines would have been spinning (Fig. 6).

**Figure 7:**
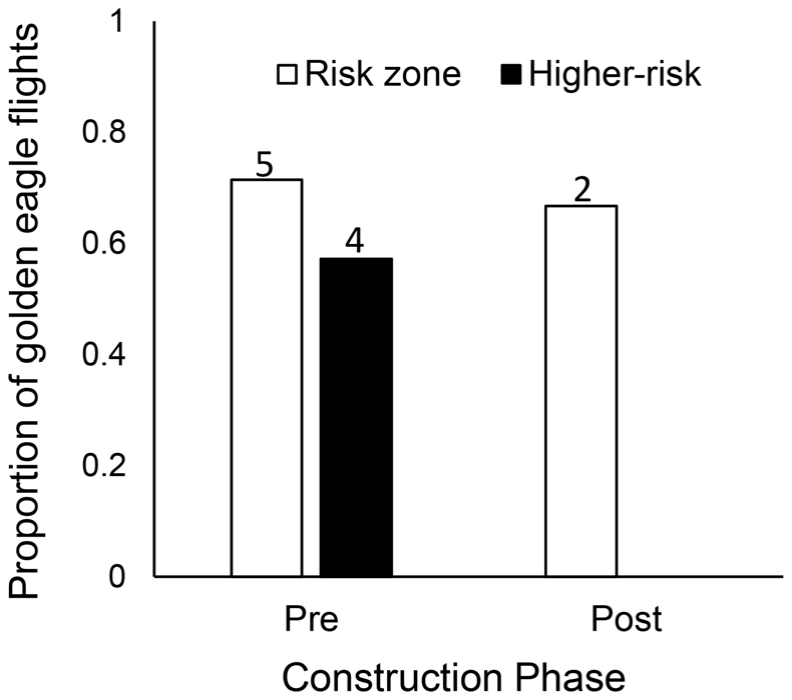
Proportion of risk zone and higher-risk crosses under tailwinds. Proportion of golden eagle ridge-top crosses within risk zone (≤150 m above ground) and of higher-risk (within risk zone at winds above turbine cut-in speed 6.8 km/h) under tailwind conditions during pre- and post-construction. Values above bars represent sample size.

In contrast, during post-construction, flights into the risk zone and of higher-risk under headwinds dropped to 7% (*n* = 5) and 3% (*n* = 2) respectively (Fig. 6). Despite the high proportion of entries into the risk zone under tailwinds in both pre- (71%) and post-construction (67%), the total number of flights under these conditions was very small (*n* = 7 and 3 respectively; Fig. 7) relative to the total number of ridge crosses detected. The proportion of higher-risk crosses under tailwinds, however, was higher pre- (57%) than during post-construction (0%).

## Discussion

The continued use of the Dokie 1 site by migrating golden eagles post-construction suggests that golden eagles are not widely displaced by this wind development facility, as has been found elsewhere [6], [23], [44]. We observed no decline in abundance and the higher number of eagles detected during post-construction may be explained by differences in local and large-scale weather phenomena [13], [45]–[49], which is beyond the scope of this study. The proportion of eagle movements over the ridge-top area, however, was similar between years, suggesting that birds are not actively avoiding the ridge-top development. The high visual acuity of golden eagles suggests that eagles likely detect the wind-energy development well before reaching the site, and thus have the ability to adjust their flight path so as to avoid the development [50].

Furthermore, the significant decrease of ridge-top crosses at rotor-swept height (risk zone; ≤150 m ag) post construction strongly suggests that when visibility is good eagles are able to adjust their flight altitudes so as to avoid closely approaching turbines. This smaller proportion of flights into the risk zone coincides with higher flight altitudes observed during post-construction, while controlling for height variation due to wind speed and wind direction. This further suggests turbine detection and avoidance by eagles. A similar finding of increased flight altitudes of raptors upon exiting a wind development in Spain has been documented [29].

Despite higher flight altitudes post-construction, there was increased probability of flights into the risk zone under head- and tailwinds compared to western crosswinds, while accounting for construction phase and wind speed. This highlights the importance of wind direction on golden eagle collision potential. Headwinds appear to pose the greatest collision risk potential, as winds under this direction are usually sufficient for the spinning of turbine blades. Tailwind conditions in our sites are typically associated with wind speeds below turbine cut-in speed, but could still constitute some risk if migrating eagles are being drawn to underlying topography in search of thermals, as found for griffon vultures in Spain [4]. Fortunately, tailwind conditions, and eagle movements under these conditions, are not common during fall migration and represent a very small number of observed flight paths. Spatial identification of concentrated crossing points under headwind and/or tailwind conditions may also allow for strategic idling of individual turbines under such conditions, and thus reducing collision risk.

Our findings suggest that migrating eagles avoided turbines during ridge-top crosses using slight adjustment of flight altitude, instead of adjusting flight trajectories around the turbine string (i.e., along windward slopes or around the ends of the turbine string). This means that collision risk may be lower for developments that fall along migratory corridors used by golden eagles compared to areas where birds spend significantly more time with repeated movements (e.g., breeding or wintering areas, where higher-risk behaviour such as aerial displays and hunting are more common) [7], [8].

While construction of wind developments along migration corridors may pose lower collision risk potential than construction in wintering/breeding areas, micro-siting of turbines within such sites may still be a significant factor in mitigating collisions [30]. For example, at the Dokie 1 site, golden eagle migration is spatially-associated with the steep windward-facing slopes where orographic lift is available [52], where turbine placement was set back slightly from these slopes on the ridge’s plateau. However, collision potential is also site-specific and can vary seasonally [3], [4], [7], [9], [51]. Initial raptor surveys early in the site planning phase can aid in identifying locations of higher collision risk potential where topography may funnel migrating raptors [9] and help in considerations of turbine siting [30].

Currently, cumulative impacts of increased wind-energy developments along this golden eagle migration corridor are poorly understood. Our study suggests that migrating golden eagles avoid turbine strings situated on a ridge top; this appears to be facilitated by increasing flight altitude prior to turbine crossing instead of a changing their migration route. The accumulated energetic costs and ability for eagles to make such fine-scale adjustments, particularly under varying weather conditions, in the face of increasing wind development along these corridors has yet to be determined.
